# Comparison of extracellular vesicles carrying bacterial DNA in urine and serum from a Korean population

**DOI:** 10.3389/fmicb.2025.1616528

**Published:** 2025-08-12

**Authors:** Ga-Eun Yie, Nam-Eun Kim, Sang-Cheol Park, Kangjin Kim, Sangah Shin, Seung Ku Lee, Chol Shin, Kwang Jun Lee, Sungho Won

**Affiliations:** ^1^Interdisciplinary Program of Bioinformatics, Seoul National University, Seoul, Republic of Korea; ^2^Institute of Health and Environment, Seoul National University, Seoul, Republic of Korea; ^3^Institute for Biomaterials, Korea University, Seoul, Republic of Korea; ^4^Department of Applied Statistics, Gachon University, Seongnam, Republic of Korea; ^5^Department of Food and Nutrition, Chung-Ang University, Anseong, Republic of Korea; ^6^Institute of Human Genomic Study, College of Medicine, Korea University, Seoul, Republic of Korea; ^7^Division of Zoonotic and Vector Borne Diseases Research, Center for Infectious Disease Research, National Institute of Health, Korea Disease Control and Prevention Agency, Cheongju, Republic of Korea; ^8^RexSoft Corp, Seoul, Republic of Korea

**Keywords:** extracellular vesicle, urine, serum, gut dysbiosis, microbiome

## Abstract

**Introduction:**

Bacteria-derived extracellular vesicles (BEVs) are emerging as key biomarkers of host-microbiota interactions. However, little is known about how BEV profiles differ across different biofluids or how these differences relate to clinical phenotypes. We aimed to examine the BEV distribution and site-specific and shared associations with host phenotypes, and evaluated the clinical relevance of microbial distance between sampling sites.

**Methods:**

We profiled BEVs using 16S rRNA sequencing of urine and serum samples from middle-aged and older Koreans (*n* = 2,827). We compared the alpha and beta diversities between the two biofluids, and assessed their relative abundances and associations with host anthropometric measurements, blood tests, and dietary nutrient intake. We also calculated the distances between urine and serum BEV compositions and assessed their clinical and metabolic implications.

**Results:**

Urine BEVs exhibited higher alpha diversity than the serum BEVs, as well as stronger associations with dietary nutrient intake, particularly sugar, and with anthropometric measures such as waist circumference. The correlations between the urine and serum BEV compositions were generally low, emphasizing their distinct microbial profiles. Notably, individuals with shorter urine-serum BEV composition distances had higher waist-to-hip and sugar-to-fat ratios.

**Discussion:**

This study provides a comprehensive comparison of urinary and serum BEVs, revealing the differences in microbial composition and associations with host phenotypes. In particular, urine BEVs showed stronger associations with dietary and metabolic factors, underscoring their potential utility as non-invasive biomarkers for metabolic health.

## 1 Introduction

The human body harbors a diverse array of microbes on its skin and mucosal surfaces. These microbial communities have been extensively studied for their associations with various diseases, clinical conditions, and dietary intake (Cuevas-Sierra et al., 2019; Manor et al., 2020; Ross et al., 2024). Traditional microbiome analyses largely focus on bacterial cells and their genomes, including those of non-viable or inactive cells (Emerson et al., 2017). However, direct interactions between microbes and host cells are limited by the epithelial barriers in the gut and other mucosal surfaces. Instead, communication between the microbiota and host occurs primarily through bacteria-derived components, such as extracellular vesicles (EVs) (Liu et al., 2024).

Bacteria-derived EVs are membrane-enclosed particles that carry cargo such as nucleic acids, proteins, lipids, and small molecules. These vesicles can traverse the intestinal epithelium and enter the bloodstream via paracellular transport, particularly under conditions of gut dysbiosis (Chakaroun et al., 2020) or through endocytosis, transcytosis, and exocytosis (Díaz-Garrido et al., 2021). Recent studies have highlighted the roles of BEVs in various diseases and clinical conditions, suggesting that they may serve as both biomarkers and mediators of host-microbiota interactions (Liu et al., 2024). BEVs isolated from urine or serum have shown promise for the detection of cancers (Kim et al., 2020; Ou et al., 2023; Park et al., 2021; Yoon et al., 2023). They have also been associated with metabolic disorders such as obesity and type 2 diabetes, supporting their influence on insulin sensitivity, inflammation, and glucose or lipid metabolism (Lee et al., 2024; Nah et al., 2019). Notably, macro- and micronutrients can regulate the production of BEVs, and high-fat or high-carbohydrate diets have been linked to gut dysbiosis and altered BEV profiles (Chae et al., 2024; Taitz et al., 2023).

Bacteria-derived EVs can be isolated from biofluids such as urine and serum, which are easily obtainable in clinical settings. While serum and urine BEVs may share certain characteristics owing to the filtration of blood into urine, they also exhibit site-specific characteristics. A previous study reported distinct bacterial compositions among the stool microbiota and BEVs from stool, serum, and urine (Nah et al., 2019). This study also observed that correlations between microbial profiles in different biofluids were higher in patients with type 2 diabetes, suggesting that investigating the associations between host phenotypes and biofluid-specific BEV abundance or microbial distance across sampling sites may provide meaningful insights into host-microbiota interactions.

In this study, we aimed to (1) investigate and compare the distribution of BEVs in serum and urine using a large cross-sectional Korean study; (2) explore site-specific and shared associations between BEVs and host phenotypes, including anthropometric measures, blood measures, and nutrient intake; and (3) assess the clinical significance of the microbial distance between sampling sites.

## 2 Materials and methods

### 2.1 Study population

The Ansan cohort is a longitudinal study of Koreans that is part of the Korean Genome Epidemiology Study (Kim et al., 2016). Participants aged ≥ 40 years were recruited from Korea University Ansan Hospital, where they completed a questionnaire, underwent anthropometric measurement, and provided blood and urine samples for clinical evaluation. Further details of this cohort are available elsewhere (Kim et al., 2016). Of the 5,012 participants, 3,879 provided urine samples and 4,779 provided serum samples for BEV composition analysis at baseline (2001–2002).

### 2.2 EV isolation and 16S rRNA gene sequencing

For EV isolation, samples were subjected to differential centrifugation at 10,000 × *g* and 4°C for 10 min using a microcentrifuge (Labogene 1730R; Bio-Medical Science, Seoul, Korea) (Lee et al., 2007). This step removes most host cells and associated intracellular components, minimizing the likelihood of host-derived bacterial DNA contamination. The supernatant was passed through a 0.22-μm filter (Inchpor2 Syringe Filter; Inchemtec, Seoul, Korea) to remove bacteria, foreign particles, and waste. The isolated EVs were boiled at 100 °C for 40 min and centrifuged at 18,214 × *g* for 30 min at 4°C to eliminate floating particles and impurities. The resulting supernatant was used for DNA extraction with a PowerSoil^®^ DNA Isolation Kit (MO BIO Laboratories, Carlsbad, CA, USA), following the manufacturer’s protocol. DNA was quantified using a QIAxpert system (Qiagen, Hilden, Germany).

Paired-end sequencing of the V3–V4 region of the bacterial 16S rRNA gene was conducted at MD Health Care (Seoul, Korea) with the MiSeq Reagent Kit v3 (600 cycles, Illumina, San Diego, CA, USA) using the widely used primers 16S_V3_F (5′ -TCGTCGGCAGCGTCAGATGTGTATAAGAGACA-GCCTACG GGNGGCWGCAG-3′) and 16S_V4_R (5′-GTCTCGTGGGCT CGGAGATGTGTATA-AGAGACAGGACTACHVGGGTATCTAA TCC-3′). Adaptor sequences were detected and removed using the CUTADAPT software,^1^ with a minimum overlap of 11, a maximum error rate of 10%, and a minimum length of 10 (Martin, 2011). Sequences were merged using CASPER,^2^ with a mismatch ratio of 0.27 and filtered based on the Phred (Q) score, resulting in sequences 350–550 bp in length (Bokulich et al., 2013; Kwon et al., 2014). After dereplication of the merged sequences, chimeric sequences were detected and removed using VSEARCH^3^ and the Silva Gold reference database for chimeras (Rognes et al., 2016). Open-reference operational taxonomic unit (OTU) picking was conducted based on the EzTaxon database using UCLUST^4^ (Edgar, 2010; Yoon et al., 2017). Samples with a read count ≤ 3,000 were excluded, leaving 3,595 urine and 3,862 serum samples. In total, 2,827 participants had both urinary and serum microbiome data (Supplementary Figure 1).

### 2.3 Clinical outcomes

Anthropometric measurements, blood biochemical tests, and questionnaires were administered at baseline for each participant. Only variables with fewer than 10% missing values were included; details with abbreviations and units are provided in Supplementary Table 1. In total, 75 variables were available, including 16 variables from anthropometric measurements, 29 from blood measurements, and 30 from dietary nutrients.

Anthropometric measurements included body composition variables measured using multi-frequency bioelectrical impedance analysis (InBody version 3.0; InBody, Seoul, Republic of Korea). For dietary nutrient variables, daily energy and nutrient intakes were calculated using a validated 103-item semi-quantitative food frequency questionnaire. The daily nutrient intake was adjusted for total energy intake using the residual method (Willett and Stampfer, 1986).

### 2.4 Covariates

Lifestyle variables including smoking, drinking, physical activity, and dietary factors were used as covariates. The intensity of each variable was calculated using a self-reported questionnaire. For smoking, pack-years were calculated by multiplying the number of packs smoked per day by the number of years of smoking. Drinking intensity was measured in grams of ethanol per day, calculated by multiplying the frequency and amount of alcohol consumption by the ethanol content. Physical activity was quantified as metabolic equivalent of task min/day. Dietary nutrient intake included a large number of variables, and factor analysis and clustering were performed to reduce the dimensionality of dietary nutrient intake. Five-factor scores, based on energy-adjusted nutrient intake, and dietary supplement intake status, were used for K-prototype clustering, which incorporated both categorical and continuous variables. The silhouette score indicated that five clusters provided the best fit.

### 2.5 Alpha and beta diversities

Alpha-diversity metrics were evaluated using observed OTUs, Chao1, and Shannon indices, and compared across sampling sites using the Wilcoxon rank-sum test. Beta diversity was assessed based on the Bray–Curtis distance after read number normalization by rarefaction, and a principal coordinate analysis plot was generated. The distance between the urine and serum samples was calculated using common OTUs found at both sample sites.

### 2.6 Permutational multivariate analysis of variance

Permutational multivariate analysis of variance was performed on the clinical variables using the Bray–Curtis distance, with 1,000 permutations (adonis function in R). Multiple comparisons were controlled using the Benjamini-Hochberg FDR (BH) method within each category (anthropometric measurements, blood measurements, and dietary nutrients). Additionally, to examine the potential influence of host conditions, PERMANOVA was also conducted based on self-reported comorbidities and current medication use, including urinary tract infection, gastric ulcer/gastritis, inflammatory or metabolic disease and drugs for metabolic conditions.

### 2.7 Microbial clusters

Microbial clusters were identified by applying the Dirichlet multinomial mixture model (Holmes et al., 2012) to the genus-level abundance profile, resulting in four microbial clusters in the urine and serum samples. To evaluate whether the microbiome clusters had a significant association with clinical variables, we conducted an *F*-test to compare the two linear regression models. In the reduced model, we included age, sex, alcohol consumption, smoking, and dietary clusters, whereas in the full model, we included microbiome clusters as additional variables.

### 2.8 Association analyses of OTUs

Association analysis was conducted using OTUs with a mean relative abundance (MRA) > 0.001 across all subjects. To robustly identify the genera associated with clinical variables, we employed two statistical tools: the phylogenetic Tree-based Microbiome Association Test (TMAT) (Kim et al., 2019) and the Microbiome Multivariable Associations with Linear Models (MaAslin2) (Mallick et al., 2021) as a validation tool. We adjusted for age, sex, smoking, drinking, physical activity, and dietary clusters (for non-dietary outcomes). Multiple comparisons were controlled using the BH method. Associations were considered significant if they had a TMAT *q* < 0.05, and showed the same direction of association in MaAslin2 (*p* < 0.05). We initially explored associations with all clinical outcomes. Additionally, we analyzed the waist to hip circumference ratio (WHR) and sugar to fat intake ratio (SFR), which are derived variables of waist circumference (WC) and sugar intake that showed consistently significant associations in prior PERMANOVA and microbial cluster analyses.

### 2.9 Correlation between urine and serum microbiome and its effect on clinical variables

Major taxa were defined as those present in > 45% of the participants with a MRA greater than 1%. For these major taxa, Spearman correlation coefficients were calculated between paired urine and serum samples using log counts-per-million (CPM) transformed values of common major taxa.

Next, we assessed the effect of the distance between the urine and serum BEVs on clinical variables. The Bray–Curtis distance was computed for each participant, and participants were divided into two groups: those with close distances (≤ first quintile, 0.908) and those with greater distances. WC, sugar intake, WHR and SFR were selected as clinical variables of interest. Differences in clinical variables between the two groups were assessed using the Wilcoxon rank-sum test.

### 2.10 Metagenome functional pathway prediction and association analysis with WHR and SFR

We used PICRUSt2 (Douglas et al., 2020) to predict functional metagenomic pathways from urine 16S rRNA data based on the Kyoto Encyclopedia of Genes and Genomes database (level 3). Pathways present by less than half of the participants and with a relative abundance < 0.05% were excluded.

Associations between predicted pathways and WHR, SFR, and the distance between urine and serum microbiome composition (≤ 0.9 or > 0.9) were assessed using a logistic regression model fitted to log CPM of pathway abundances. Adjustments were made for age, sex, drinking status, smoking status, physical activity, and dietary clusters (excluding the dietary cluster for the SFR). Multiple comparisons were controlled using the BH method.

## 3 Results

### 3.1 Baseline characteristics

The baseline characteristics of the study participants are shown in Supplementary Table 2. The analysis included 2,827 participants with both urine and serum microbiome data. The average age of the participants was 48.79 years (standard deviation, SD = 7.65), and 46.9% were males. The mean daily energy intake was 1,979.13 kcal. The average body mass index was 24.71 kg/m^2^ (SD = 2.98), and the WC was 80.54 cm (SD = 8.61). The mean values of the clinical variables were within normal ranges. Only 19 participants (0.67%) reported a history of urinary tract infection, and 16.8% were currently taking at least one medication regardless of type. BEV composition did not significantly differ by most of the self-reported comorbidities or current medication use (Supplementary Table 3).

### 3.2 Comparison of BEVs in urine and serum

**FIGURE 1:**
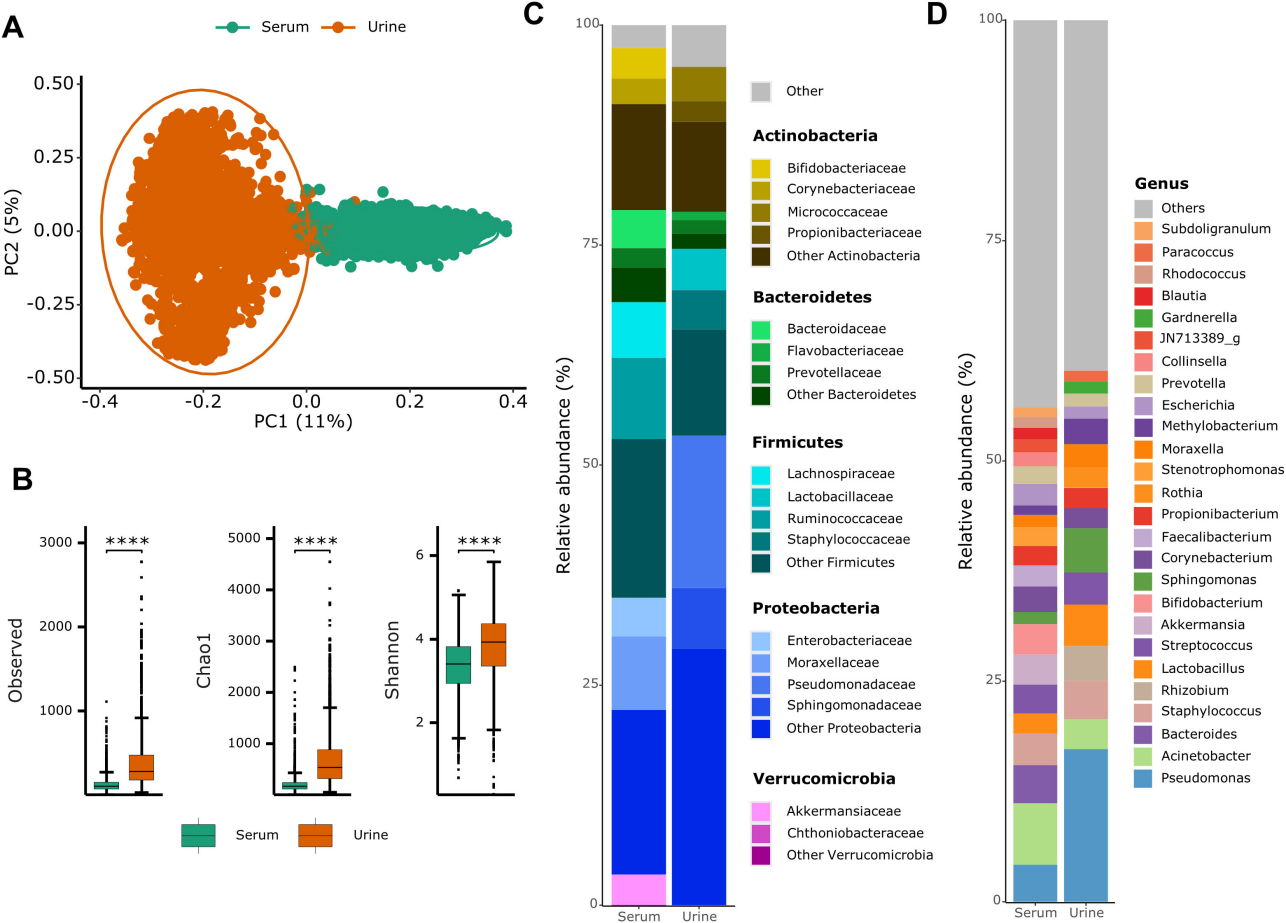
Comparison of bacteria-derived extracellular vesicles between urine and serum samples. **(A)** Beta diversity based on Bray–Curtis distance and **(B)** alpha diversity plots for both urine and serum samples. ****p < 0.0001. **(C)** Phylum- and **(D)** genus-level relative abundance bar plots by sampling site.

Beta diversity analysis (Bray–Curtis distance) revealed that urine and serum BEV microbiomes were clearly distinct (Figure 1A). Additionally, urine BEVs were significantly more diverse than serum BEVs across all three alpha diversity measures: observed OTUs, Chao1, and Shannon index (Figure 1B). Relative abundance plots at the phylum and genus levels were generated, displaying the top 20 genera with MRA > 0.01 in either urine or serum, while lower abundance genera were grouped under “Other.” At the phylum level, Proteobacteria, Firmicutes, Bacteroidetes, and Actinobacteria were common taxa with high MRAs at both sampling sites (Figures 1C, D). Notably, urine exhibited a higher MRA of the Proteobacteria; specifically, an elevated presence of the *Pseudomonas* genus within this phylum.

### 3.3 Correlation of common major taxa between sampling sites

Sixteen genera in urine and 21 in serum were present in over 45% of participants with MRA > 0.01. Of these, 12 genera, including *Acinetobacter, Corynebacterium, Escherichia, Lactobacillus, Methylobacterium, Moraxella, Prevotella, Propionibacterium, Pseudomonas, Sphingomonas, Staphylococcus*, and *Streptococcus* were shared between the sample types (Table 1). Spearman’s correlation coefficients were calculated between urine and serum samples for these common major genera. Among the genera examined, *Staphylococcus, Pseudomonas*, and *Escherichia* showed correlations of 0.05, −0.05 and −0.09, respectively, with only *Escherichia* showing a significant correlation after correction for multiple testing.

**TABLE 1:** Major genera and their correlations between sampling sites.

Phylum	Family	*Genus*	Urine	Serum	Spearman correlation^a^	*q* value
Actinobacteria	Bifidobacteriaceae	*Gardnerella*	O	–	–	–
	Bifidobacteriaceae	*Bifidobacterium*	–	O	–	–
	Coriobacteriaceae	*Collinsella*	–	O	–	–
	Corynebacteriaceae	*Corynebacterium*	O	O	−0.030	0.222
	Micrococcaceae	*Rothia*	O	–	–	–
	Nocardiaceae	*Rhodococcus*	–	O	–	–
	Propionibacteriaceae	*Propionibacterium*	O	O	−0.022	0.356
Bacteroidetes	Bacteroidaceae	*Bacteroides*	–	O	–	–
	Prevotellaceae	*Prevotella*	O	O	0.030	0.222
Firmicutes	Lachnospiraceae	*Blautia*	–	O	–	–
	Lactobacillaceae	*Lactobacillus*	O	O	0.037	0.155
	Ruminococcaceae	*Faecalibacterium*	–	O	–	–
	Ruminococcaceae	*JN713389_g*	–	O	–	–
	Staphylococcaceae	*Staphylococcus*	O	O	0.047	0.051
	Streptococcaceae	*Streptococcus*	O	O	−0.021	0.356
Proteobacteria	Enterobacteriaceae	*Escherichia*	O	O	−0.091	0.000
	Methylobacteriaceae	*Methylobacterium*	O	O	−0.005	0.794
	Moraxellaceae	*Acinetobacter*	O	O	−0.026	0.283
	Moraxellaceae	*Moraxella*	O	O	0.012	0.585
	Pseudomonadaceae	*Pseudomonas*	O	O	−0.047	0.051
	Rhizobiaceae	*Rhizobium*	O	–	–	–
	Rhodobacteraceae	*Paracoccus*	O	–	–	–
	Sphingomonadaceae	*Sphingomonas*	O	O	−0.012	0.585
	Xanthomonadaceae	*Stenotrophomonas*	–	O	–	–
Verrucomicrobia	Akkermansiaceae	*Akkermansia*	–	O	–	–

Major genera were defined as those present in > 45% of the participants with a mean relative abundance > 1%. ^a^Log counts-per-million values were used to calculate Spearman correlations.

### 3.4 Clinical variables explaining variance in BEV composition

**FIGURE 2:**
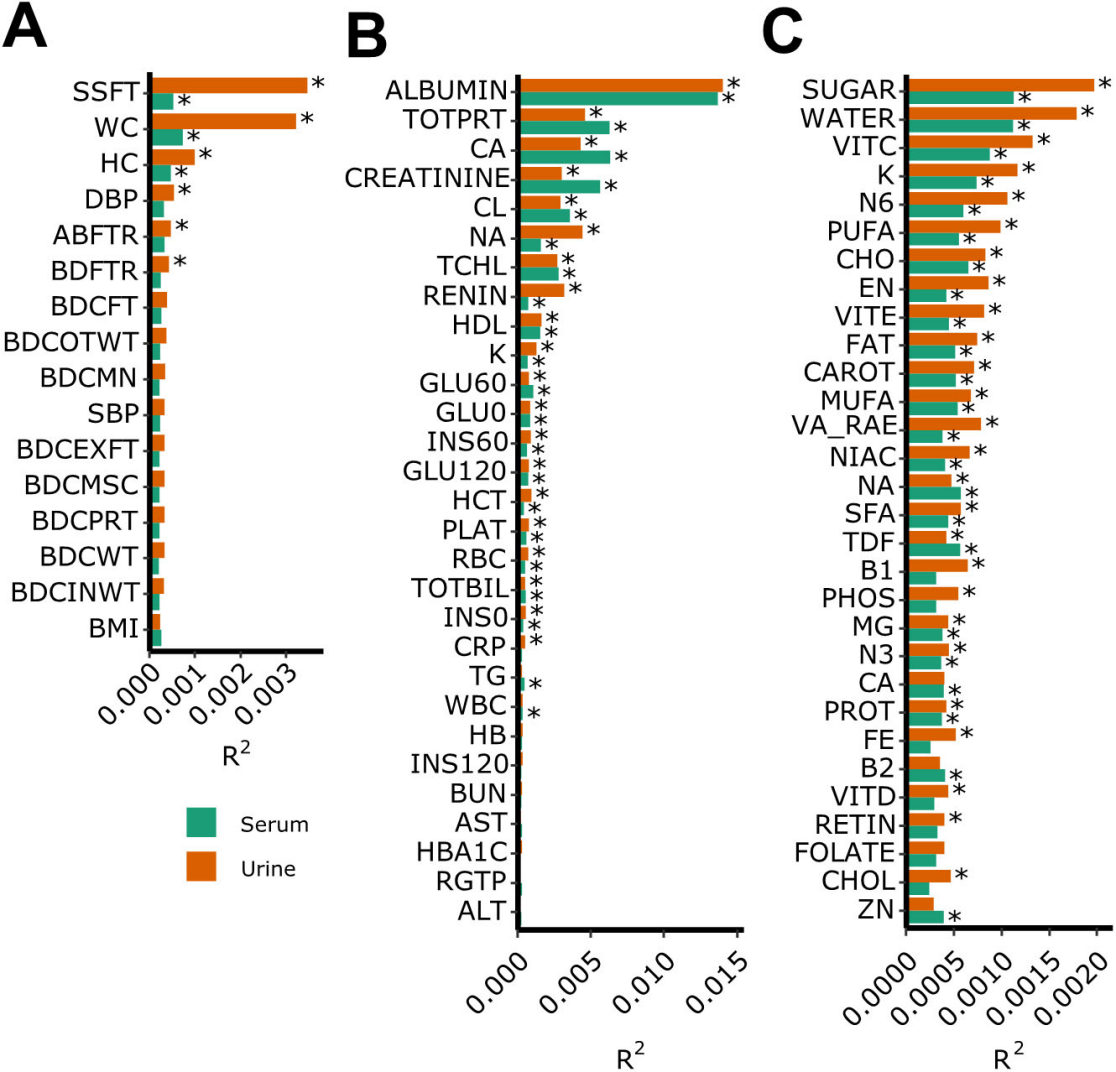
Proportion of variance in the bacteria-derived extracellular vesicle composition explained by clinical variables. **(A)** Anthropometric measurement, **(B)** blood measurement, and **(C)** dietary nutrients intake. *q < 0.05.

Figure 2 illustrates the proportion of variance (R^2^) in the BEV composition explained by various clinical variables, including anthropometric measurements, blood measurements, and dietary intake of nutrients. Among the anthropometric measurements (Figure 2A), WC, hip circumference, and subscapular skinfold thickness explained the highest R^2^ in both serum and urine samples, with the associations generally stronger in urine. Regarding the blood test results (Figure 2B), albumin, total protein, and calcium levels explained the greatest proportion of variance in the BEV composition, showing stronger effects in the serum samples. Regarding dietary nutrient intake (Figure 2C), sugar, water, vitamin C, potassium, and N-6 fatty acid intakes were most strongly associated with variance in BEV, with urine showing a slightly stronger relationship. Notably, in both dietary nutrients and anthropometric measurements, the urine BEV composition consistently showed stronger associations with clinical variables than the serum BEV composition.

### 3.5 Microbiome clusters and their related clinical characteristics

**FIGURE 3:**
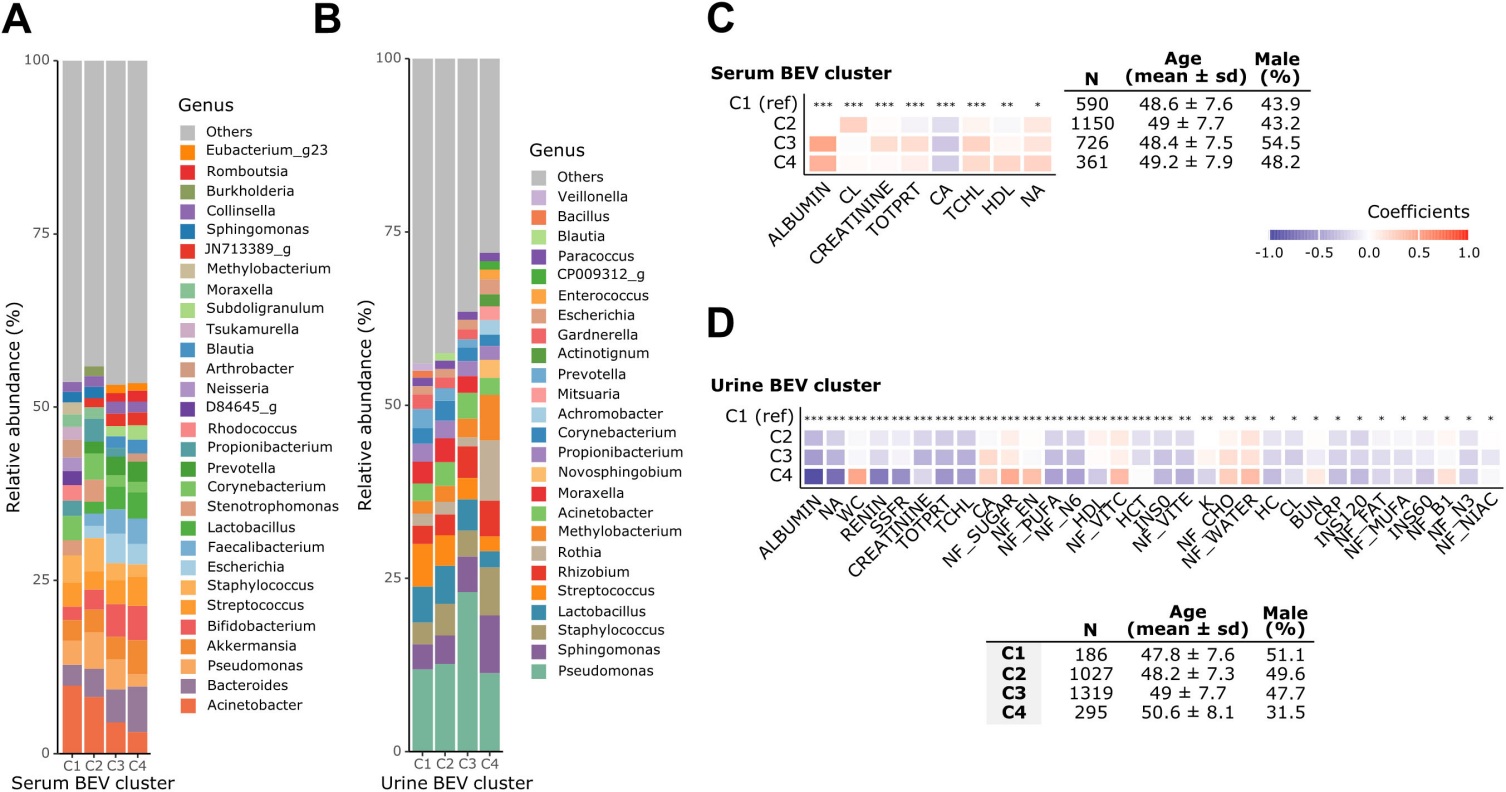
Microbiome clusters and their associated clinical characteristics. Relative abundances of genera in **(A)** serum and **(B)** urine bacteria-derived extracellular vesicle (BEV) clusters. Clinical characteristics associated with **(C)** serum and **(D)** urine BEV clusters. Adjusted for age, sex, alcohol consumption, smoking, and dietary clusters for non-nutrient variables. *q < 0.05, **q < 0.01, ***q < 0.001.

To explore overall patterns in BEV composition prior to genus-level analysis, we identified clusters of microbiome profiles in urine and serum BEVs and examined their associations with various clinical variables, adjusting for age, sex, drinking, smoking, and dietary clusters for non-nutrient variables (Figure 3).

In the urine BEV clusters, the relative abundance of genera varied across the four distinct clusters. In cluster 4, *Sphingomonas, Staphylococcus, Rothia, Methylobacterium*, and *Novosphingobium* were dominant, whereas genera such as *Lactobacillus* and *Streptococcus* were less abundant compared to other clusters. Cluster 4 also had a greater proportion of the top 20 most prevalent genera, with lower abundance genera grouped as “Other.” Demographically, individuals in cluster 4 were older and had a higher proportion of females. Most clinical variables that were significantly associated with urine BEV clusters showed a decreasing trend toward cluster 4 when cluster 1 was used as the reference. However, WC, serum calcium level, and sugar, energy, carbohydrate, and water intake were the highest in cluster 4.

In the serum BEV clusters, the relative abundance of *Acinetobacter* decreased from cluster 1 to cluster 4, whereas *Bacteroides* exhibited the opposite pattern, increasing toward cluster 4. Compared to urine, fewer clinical variables were statistically significantly associated with serum microbiome clusters, and those that were significantly associated were predominantly blood measurement variables.

### 3.6 Association analysis

**FIGURE 4:**
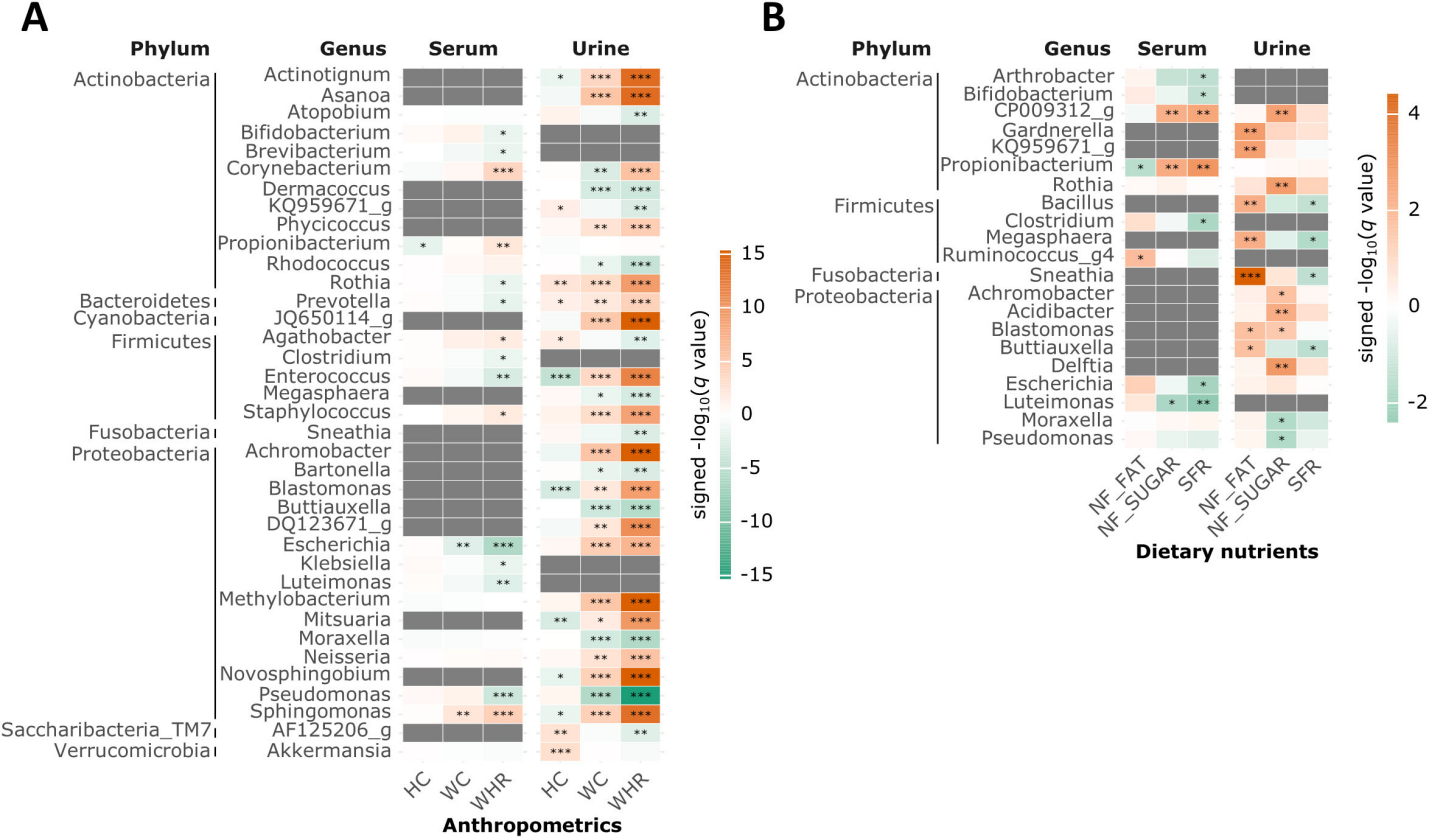
Bacteria-derived extracellular vesicles (BEVs) related to clinical variables in each sampling site. **(A)** Anthropometric measurements **(B)** dietary nutrients. A heatmap displaying values of −log (*q* value) multiplied by the sign of coefficients. BEVs showing a significant association with at least one clinical variable (Tree-based Microbiome Association Test (TMAT) *q* < 0.05 and Microbiome Multivariable Associations with Linear Models (MaAsLin2) *p* < 0.05 with same sign of coefficients) are included. Adjusted for age, sex, alcohol consumption, smoking, and dietary clusters for non-nutrient variables. Gray color indicates that the association between the BEVs and clinical variables was not analyzed, because BEVs filtered out according to the mean relative abundance. *q < 0.05, **q < 0.01, ***q < 0.001.

Figure 4 presents the BEV features that were significantly associated with the clinical variables, as identified by TMAT and further validated using MaAsLin2. Only the associations that were consistent across both methods are shown.

*Bifidobacterium* in the serum and *Megasphaera* in the urine were negatively associated with the WHR and SFR. Conversely, *Sphingomonas*, *Staphylococcus*, and *Corynebacterium* were consistently positively associated with WHR in both serum and urine. In contrast, *Rothia*, *Prevotella*, *Enterococcus*, and *Escherichia* were positively associated with WHR in urine, but negatively associated in serum, indicating sampling site-specific relationships.

Overall, dietary nutrients and anthropometric measurements were primarily associated with urine BEVs, whereas blood measurements showed a greater number of associations with BEV features in both serum and urine (Supplementary Figure 2). These results suggest that dietary and anthropometric factors were more closely linked to the urine microbiome, whereas blood-related variables exhibited broader connections across both sampling sites.

### 3.7 Clinical variables associated with the distance between urine and serum BEV composition

**FIGURE 5:**
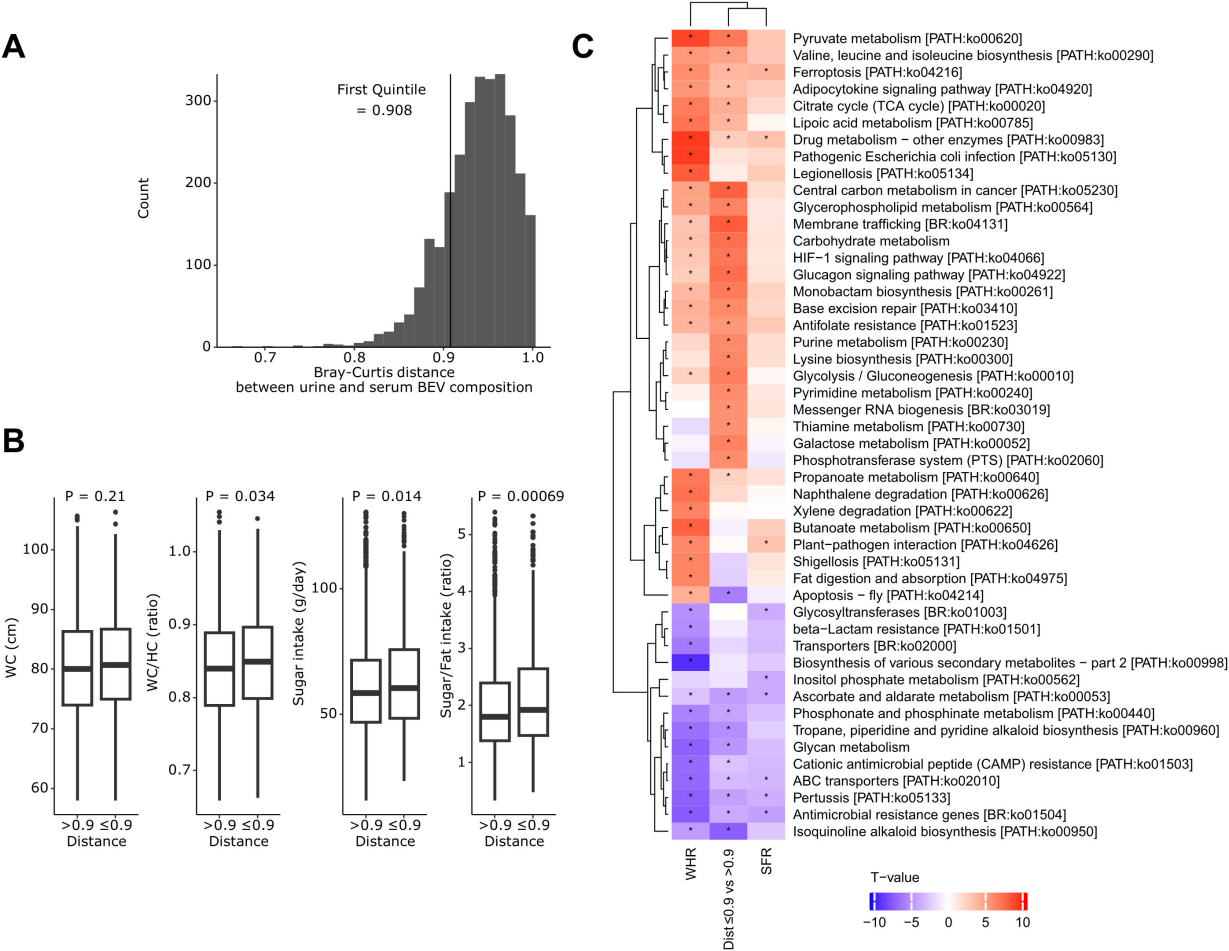
Clinical variables and predicted pathways associated with the distance between urine and serum bacteria-derived extracellular vesicle composition. **(A)** Distribution of Bray–Curtis distances between urine and serum BEV compositions. **(B)** Comparison of clinical variables between participants with close distances (≤ first quintile, 0.908) and those with greater distances. *P*-values were calculated using the Wilcoxon rank-sum test. **(C)** A heatmap showing *t*-values of the association between predicted Kyoto Encyclopedia of Genes and Genomes pathways and the waist-to-hip circumference ratio (WHR), sugar-to-fat intake ratio (SFR), and distance group. *p < 0.05.

Figure 5A illustrates that the Bray–Curtis distance between urine and serum BEV composition ranged from 0.67 to 1, with most participants clustering near 1. The first quintile corresponded to a distance of 0.908. Participants with more similar BEV compositions between urine and serum (≤ 0.908) had significantly lower WHR (*p* = 0.034), daily sugar intake (*p* = 0.014), and SFR (*p* = 0.00069) compared to those with greater distance (Figure 5B). Although WC did not reach statistical significance, it showed a similar trend (*p* = 0.21).

The WHR, SFR, and urine-serum distance groups exhibited similar patterns of association with the predicted pathways (Figure 5C). Pathways that were more abundant in participants with elevated SFR, higher WHR, and closer urine-serum microbiome composition distances included those related to carbohydrate metabolism (citrate cycle, glycolysis/gluconeogenesis, pyruvate, galactose, propanoate, and butanoate metabolism), endocrine system (adipocytokine and glucagon signaling pathway), membrane trafficking, phosphotransferase system (PTS), and oxidative stress (ferroptosis, and HIF-1 signaling pathway) (*q* < 0.05).

## 4 Discussion

In this study, we examined BEVs in urine and serum samples from a large cohort of Korean adults, focusing on their composition, diversity, and associations with host dietary, anthropometric, and clinical factors. Our findings highlight the distinct differences between urine and serum BEVs, offering new insights into their biofluid-specific profiles and relationships with host phenotypes. Urine BEVs exhibited higher alpha diversity than those derived from the serum, which is consistent with reports from other East Asian populations (Park et al., 2022; Nah et al., 2019). This elevated alpha diversity in urine could stem from the broader range of vesicle origins; urine contains BEVs that originate not only from the bloodstream but also from the urogenital tract (Whiteside et al., 2015).

While most previous studies examined the association between host phenotypes and microbes using bacterial genomic data from stool or vaginal samples, our investigation focused on BEVs, revealing both consistent and novel findings. For instance, *Bifidobacterium*-derived EVs in serum and *Megasphaera*-derived EVs in urine were negatively associated with WHR, which is consistent with their previously reported roles in obesity (Gao et al., 2018; Sroka-Oleksiak et al., 2020; Lv et al., 2019). Conversely, *Staphylococcus*- and *Corynebacterium*-derived EVs in both biofluids were positively associated with WHR, which is consistent with previous findings (Sroka-Oleksiak et al., 2020; Vongsa et al., 2019). Furthermore, *Prevotella-* and *Escherichia-*derived EVs, known to increase in obesity (Gao et al., 2018; Nirmalkar et al., 2018; Thingholm et al., 2019), showed positive associations exclusively in urine, but negative associations in serum, suggesting that functional differences in BEVs across biofluids may underpin these differing relationships. Additionally, urine BEVs showed stronger associations with dietary nutrient intake, particularly sugar, and with anthropometric measurements, suggesting that urine BEVs may better reflect dietary and body composition factors. This trend was consistently observed in both microbiome cluster- and genus-level association analyses, and the coherence across analytical approaches underscores the potential utility of urine BEVs as biomarkers of host metabolic status. Further research is warranted to elucidate the biological mechanisms underlying these differential associations.

We observed a low overall correlation between urine and serum BEV compositions, highlighting their distinct microbial profiles. Individuals with closer urine-serum BEV composition distances exhibited higher WHR and SFR, suggesting that central adiposity and sugar-rich diets may coincide with greater convergence of microbial signals across serum and urine. One plausible explanation for this convergence lies in gut barrier dysfunction under metabolic stress. Metabolic stress is known to promote low-grade inflammation and compromise intestinal barrier integrity. Lipopolysaccharide, a component of the outer membrane of Gram-negative bacteria, contributes to intestinal inflammation and disrupts tight junction organization, thereby increasing gut permeability (Di Vincenzo et al., 2024). In addition, high-sugar diets have been shown to alter gut microbiota composition, reduce microbial diversity, and impair intestinal barrier function, facilitating the translocation of microbial products, including BEVs, into systemic circulation (Saffouri et al., 2019). As a result, increased BEV exposure in the bloodstream may lead to the presence of more similar BEV composition in both serum and urine. Supporting this notion, a study using a different Korean cohort found that correlations between urine and serum BEV profiles were higher in individuals with type 2 diabetes (Nah et al., 2019).

To explore these host-microbiota interactions, we predicted functional abundances using urine BEV data. Pathways related to carbohydrate metabolism, endocrine system, oxidative stress, PTS, and membrane trafficking were enriched among individuals with higher WHR, SFR, and closer serum-urine BEV distances. Carbohydrate metabolic pathways, which are involved in energy harvesting, and the PTS, a bacterial system that facilitates the transport and phosphorylation of carbohydrates (Deutscher et al., 2006), have been previously implicated in obesity and insulin resistance (Liu et al., 2017; Takeuchi et al., 2023; Turnbaugh et al., 2009; Turnbaugh et al., 2006). Enrichment of membrane trafficking pathways, which mediate the internalization and intracellular transport of EVs (Gurunathan et al., 2021), in individuals with shorter serum-urine BEV distances may reflect enhanced EV transport activity under metabolic stress. These findings support the hypothesis that metabolic conditions may promote convergence in the microbial signatures of distinct biofluids, potentially due to increased barrier permeability and systemic EV dissemination. However, these pathway predictions rely on 16S rRNA data and should be interpreted with caution until validation using multi-omics approaches.

Among anthropometric variables, those associated with abdominal obesity–including waist circumference, hip circumference, abdominal fat ratio, and subscapular skinfold thickness–explained the largest proportion of variance in BEV composition, particularly in urine samples. This reinforces the link between adiposity and microbial vesicle profiles, which has been observed not only for bacterial taxa but also at the EV level. In terms of dietary intake, sugar intake showed the highest explanatory power among nutrients. Given the high carbohydrate consumption typical among middle-aged and older Korean populations (median 74% of daily calories), our findings suggest that dietary sugar, rather than fat, may be a particularly relevant modulator of BEV composition in this context.

Regarding blood markers, albumin and calcium explained a substantial proportion of the variance in BEV profiles. Albumin is a key marker not only of age and nutritional status, but also of vesicle stability (Wolf et al., 2012). The association with serum calcium levels may reflect an underlying disruption in calcium homeostasis. Further studies are needed to better understand how laboratory biomarkers influence BEV profiles.

This study has several limitations. First, although we included available data on comorbidities and current medication use, we cannot exclude the potential influence of unmeasured confounders. In particular, a lack of information on recent antibiotic use remains a limitation. Second, experimental validation techniques commonly used in EV research (e.g., transmission electron microscopy or nano particle tracking analysis) were not performed due to the practical constraints at the time of sample processing, and contamination could not be directly assessed using negative controls. Third, despite the implementation of strict protocols to minimize contamination, the results may have been influenced by potential contamination during sample collection and processing, which is an inherent challenge for low-biomass samples (Tulkens et al., 2020). Fourth, although we referred to the EVs containing bacterial DNA as bacteria-derived EVs, we acknowledge that our isolation method, based primarily on differential centrifugation, cannot definitively distinguish bacterial EVs from human-derived EVs that may carry bacterial components. Therefore, the detected bacterial DNA signatures should be interpreted with caution, and future studies employing more rigorous EV purification and characterization techniques are warranted. Fifth, while we focused on urine and serum BEVs, comparisons with other biofluids such as saliva or stool were not possible because of the lack of corresponding data. The inclusion of additional biofluids in future studies could enhance our understanding of BEV profiles across different sample types. Sixth, the observed associations could not establish causality owing to the cross-sectional design of our study. Finally, our study population exclusively consisted of East Asian middle-aged and older adults, which potentially limit the applicability of these findings to other populations.

In conclusion, this study provides a comprehensive comparison of urinary and serum microbial signals through the analysis of BEVs, uncovering the distinct characteristics of microbial composition, diversity, and associations with host phenotypes in urine and serum. Urinary BEVs demonstrated stronger associations with dietary nutrient intake and anthropometric measurements, suggesting their potential as non-invasive biomarkers for metabolic health.

## Data Availability

The data presented in the study are deposited in the Figshare repository under the following doi: https://doi.org/10.6084/m9.figshare.29582258.

## Footnotes

1
https://cutadapt.readthedocs.io

2
http://best.snu.ac.kr/casper

3
https://github.com/torognes/vsearch

4
http://www.drive5.com/usearch
