# Supplementary material for: Comparison of extracellular vesicles carrying bacterial DNA in urine and serum from a Korean population
Source: Front Microbiol. 2025 Aug 12;16:1616528. doi: 10.3389/fmicb.2025.1616528 (PMC12379074; doi:10.3389/fmicb.2025.1616528)
Supplement: Supplementary file 1 [file Data_Sheet_1.docx]

Supplementary Material

# Supplementary Figures

**Ansan cohort**

n = 5,012

**Urine sample**

n = 3,879

**Serum sample**

n = 4,779

**Urine EV microbiome**

n = 3,595

**Serum EV microbiome**

n = 3,862

**Common**

n = 2,827

QC & read count filtering <3000

**Supplementary Figure 1.** **Flowchart of study participants**


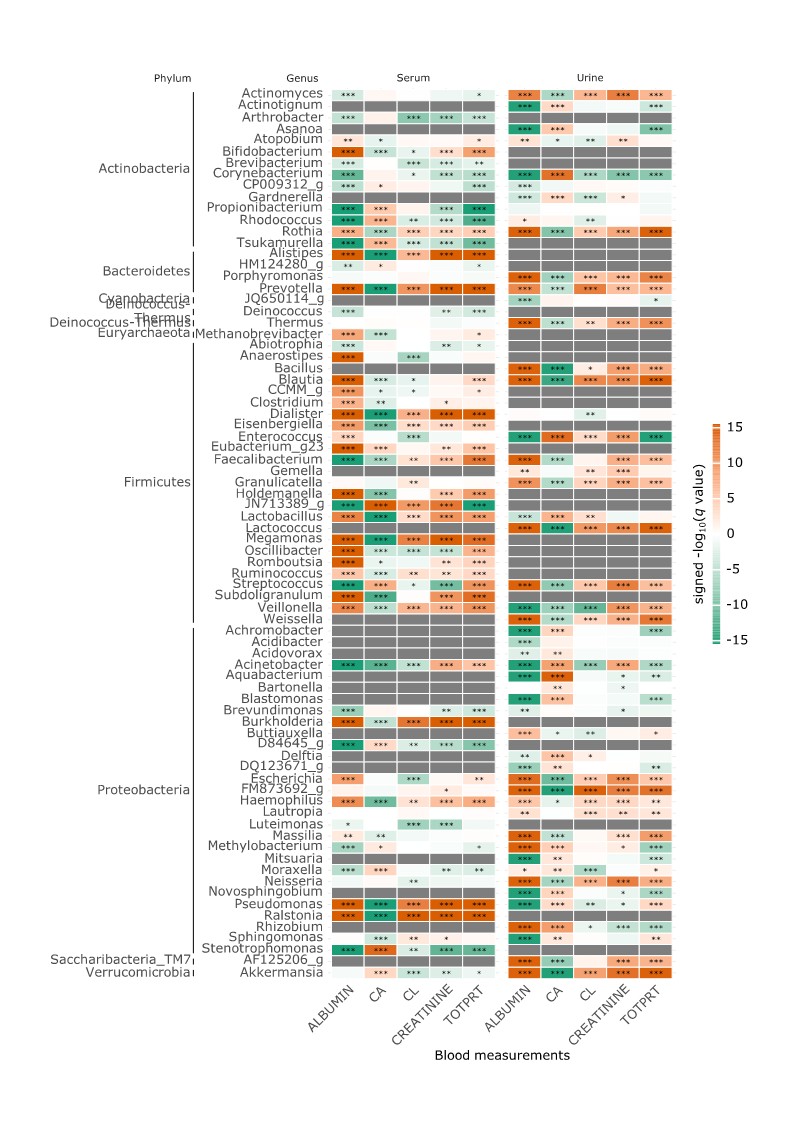


**Supplementary Figure 2. BEV related to blood measurements in each sampling site**

A heatmap displaying values of -log(*q* value) multiplied by the sign of coefficients. BEV showing significant association with at least one clinical variable (TMAT *q* value < 0.05 and MaAsLin2 *p* value < 0.05 with same sign of coefficients) are included. Adjusted for age, sex, alcohol consumption, smoking, and dietary clusters for non-nutrient variables. Gray color indicates that the association between the BEV and clinical variable was not analyzed, because BEV filtered out according to mean relative abundance. **q*< 0.05 ***q*< 0.01 ****q*<0.001

# Supplementary Tables

**Supplementary Table 1. List of clinical outcomes**

| **Groups** | **Variables** | **Unit** | **Abbreviations** |
| --- | --- | --- | --- |
| Anthropometric measurements (16) | (InBody^a^) Intracellular water | L | BDCINWT |
|  | (InBody^a^) Total body water | L | BDCWT |
|  | (InBody^a^) Muscle mass | kg | BDCMSC |
|  | (InBody^a^) Fat-free mass | kg | BDCEXFT |
|  | (InBody^a^) Extracellular water | L | BDCOTWT |
|  | (InBody^a^) Protein | kg | BDCPRT |
|  | (InBody^a^) Mineral | kg | BDCMN |
|  | (InBody^a^) Fat | kg | BDCFT |
|  | (InBody^a^) Fat ratio | % | BDFTR |
|  | (InBody^a^) Abdominal fat ratio | % | ABFTR |
|  | Body mass index | kg/m^2^ | BMI |
|  | Waist circumference | cm | WC |
|  | Hip circumference | cm | HC |
|  | Subscapular skinfold thickness | mm | SSFT |
|  | Systolic blood pressure | mmHg | SBP |
|  | Diastolic blood pressure | mmHg | DBP |
| Blood measurements (29) | Fasting blood glucose | mg/dL | GLU0 |
|  | 1-hour OGTT glucose | mg/dL | GLU60 |
|  | 2-hour OGTT glucose | mg/dL | GLU120 |
|  | Albumin | g/dL | ALBUMIN |
|  | Blood urea nitrogen | mg/dL | BUN |
|  | Creatinine | mg/dL | CREATININE |
|  | Aspartate transaminase | IU/L | AST |
|  | Alanine transaminase | IU/L | ALT |
|  | γ-glutamyl transpeptidase (GTP) | IU/L | RGTP |
|  | Total cholesterol | mg/dL | TCHL |
|  | High density lipoprotein cholesterol | mg/dL | HDL |
|  | Triglyceride | mg/dL | TG |
|  | Total protein | g/dL | TOTPRT |
|  | Total bilirubin | mg/dL | TOTBIL |
|  | Serum Calcium | mmol/L | CA |
|  | Serum Sodium | mmol/L | NA |
|  | Serum Potassium | mmol/L | K |
|  | Serum Chloride | mmol/L | CL |
|  | C-Reactive protein | mg/dL | CRP |
|  | White blood cells | 10^3^/µL | WBC |
|  | Red blood cells | 10^6^/µL | RBC |
|  | Hemoglobin | g/dL | HB |
|  | Hematocrit | % | HCT |
|  | HbA1C | % | HBA1C |
|  | Platlet | 10^3^/µL | PLAT |
|  | Fasting insulin | µIU/ml | INS0 |
|  | 1-hour OGTT insulin | µIU/ml | INS60 |
|  | 2-hour OGTT insulin | µIU/ml | INS120 |
|  | Renin | ng/mL/hr | RENIN |

^a^ InBody version 3.0; InBody, Seoul, Republic of Korea

**Supplementary Table 1. List of clinical outcomes (*continued*)**

| Groups | Variables | Unit | Abbreviations |
| --- | --- | --- | --- |
| Dietary nutrients (30) | Energy | kcal | NF_EN |
|  | Water | g | NF_WATER |
|  | Protein | g | NF_PROT |
|  | Fat | g | NF_FAT |
|  | Saturated fatty acids | g | NF_SFA |
|  | Monounsaturated fatty acids | g | NF_MUFA |
|  | Polyunsaturated fatty acids | g | NF_PUFA |
|  | N-3 fatty acids | g | NF_N3 |
|  | N-6 fatty acids | g | NF_N6 |
|  | Cholesterol | mg | NF_CHOL |
|  | Carbohydrate | g | NF_CHO |
|  | Total dietary fiber | g | NF_TDF |
|  | Sugar | g | NF_SUGAR |
|  | Calcium | mg | NF_CA |
|  | Phosphorus | mg | NF_PHOS |
|  | Sodium | mg | NF_NA |
|  | Potassium | mg | NF_K |
|  | Magnesium | mg | NF_MG |
|  | Iron | mg | NF_FE |
|  | Zinc | mg | NF_ZN |
|  | Vitamin A | µg RAE | NF_VA_RAE |
|  | Vitamin D | µg | NF_VITD |
|  | Vitamin E | mg α-TE | NF_VITE |
|  | Beta-carotene | µg | NF_CAROT |
|  | Retinol | µg | NF_RETIN |
|  | Vitamin B1 | mg | NF_B1 |
|  | Vitamin B2 | mg | NF_B2 |
|  | Niacin | mg | NF_NIAC |
|  | Folate | µg DFE | NF_FOLATE |
|  | Vitamin C | mg | NF_VITC |

**Supplementary Table 2. Baseline characteristics of study participants**

|  | Ansan cohort (n=2,827) |
| --- | --- |
| Age (years) | 48.79 (7.65) |
| Sex |  |
| Male | 1326 (46.9%) |
| Female | 1501 (53.1%) |
| Drinking (g/day) | 10.46 (23.54) |
| Smoking (pack-years) | 8 (14.3) |
| Physical activity (METs) | 582.08 (1029.1) |
| Energy intake (kcal/day) | 1979.13 (569.05) |
| Sugar intake (energy adjusted g/day) | 62.76 (22.72) |
| Dietary cluster |  |
| C1 | 786 (28.08%) |
| C2 | 541 (19.33%) |
| C3 | 609 (21.76%) |
| C4 | 210 (7.5%) |
| C5 | 653 (23.33%) |
| BMI (kg/m2) | 24.71 (2.98) |
| WC (cm) | 80.54 (8.61) |
| HC (cm) | 95.44 (4.99) |
| SBP (mmHg) | 116.9 (17.14) |
| DBP (mmHg) | 77.79 (11.65) |
| GLU0 (mg/dL) | 87.33 (23.25) |
| HBA1C (%) | 5.73 (0.87) |
| TG (mg/dL) | 163.11 (108.18) |
| TCHL (mg/dL) | 191.83 (34.37) |
| HDL (mg/dL) | 44.14 (9.61) |
| Albumin (g/dL) | 4.24 (0.29) |
| Disease history (yes, %) |  |
| Urinary tract infection | 19 (0.67%) |
| Gastric ulcer/gastritis | 543 (19.21%) |
| Arthritis | 314 (12.62%) |
| Asthma | 47 (1.66%) |
| Allergy | 122 (4.32%) |
| Hypertension | 340 (12.03%) |
| Diabetes | 153 (5.41%) |
| Dyslipidemia | 97 (3.43%) |
| Tumor^a^ | 73 (2.58%) |
| Current medication usage (yes, %) |  |
| Any medication | 475 (16.80%) |
| Metabolic disease medication^b^ | 253 (8.95%) |

Data are presented as mean (standard deviation) or N (%).

^a^Tumor includes both benign and malignant cases.

^b^Metabolic disease include hypertension, diabetes, and dyslipidemia.

**Supplementary Table 3. Permutational multivariate analysis of variance results of self-reported disease history and current medication use**

|  | Serum | |  | Urine | |
| --- | --- | --- | --- | --- | --- |
| Variables | R^2^ | *p* value |  | R^2^ | *p* value |
| Disease history |  |  |  |  |  |
| Urinary tract infection | 0.0003 | 0.898 |  | 0.0003 | 0.451 |
| Gastric ulcer/gastritis | 0.0003 | 0.896 |  | 0.0006 | 0.009 |
| Arthritis | 0.0004 | 0.089 |  | 0.0003 | 0.663 |
| Asthma | 0.0003 | 0.839 |  | 0.0003 | 0.634 |
| Allergy | 0.0004 | 0.217 |  | 0.0006 | 0.011 |
| Hypertension | 0.0003 | 0.551 |  | 0.0002 | 0.999 |
| Diabetes | 0.0002 | 0.994 |  | 0.0004 | 0.407 |
| Dyslipidemia | 0.0003 | 0.817 |  | 0.0004 | 0.326 |
| Tumor^a^ | 0.0004 | 0.393 |  | 0.0003 | 0.434 |
| Current drug usage |  |  |  |  |  |
| Any medication | 0.0004 | 0.485 |  | 0.0003 | 0.634 |
| Metabolic disease medication^b^ | 0.0003 | 0.522 |  | 0.0003 | 0.876 |

^a^Tumor includes both benign and malignant cases.

^b^Metabolic disease include hypertension, diabetes, and dyslipidemia.
